# Family centeredness of care: a cross-sectional study in intensive care units part of the European society of intensive care medicine

**DOI:** 10.1186/s13613-024-01307-0

**Published:** 2024-05-21

**Authors:** Élie Azoulay, Nancy Kentish-Barnes, Carole Boulanger, Giovanni Mistraletti, Margo van Mol, Gabriel Heras-La Calle, Elisa Estenssoro, Peter Vernon van Heerden, Maria-Cruz Martin Delgado, Anders Perner, Yaseen M Arabi, Sheila Nainan Myatra, Jon Henrik Laake, Jan J. De Waele, Michael Darmon, Maurizio Cecconi

**Affiliations:** 1 Médecine Intensive et Réanimation, APHP, Hôpital Saint-Louis, Paris-Cité University, 1 avenue Claude Vellefaux, Paris, 75010 France; 2 Royal Devon University NHS Foundation Trust, Barrack Road, Exeter, UK; 3 Dipartimento di Fisiopatologia medico-chirurgica e dei trapianti. A.S.S.T. Ovest Milanese, Università degli Studi di Milano, Ospedale Civile di Legnano, Legnano, MI Italy; 4 Erasmus MC, Rotterdam, Netherlands; 5 International Research Project for the Humanisation of Intensive Care Units, Proyecto HU-CI, Madrid, Spain; 6 Humanizing Healthcare Foundation. Intensive Care Unit, Hospital Universitario de Jaén, Jaén, Spain; 7 Hospital Interzonal de Agudos General San Martín, La Plata, Buenos Aires, Argentina; 8 Department of Anesthesiology, Critical Care and Pain medicine, Faculty of Medicine, Hadassah Medical Center, Hebrew University of Jerusalem, Jerusalem, Israel; 9 Department Intensive Care Medicine Hospital 12 de Octubre, Madrid, Spain; 10 Research Institute “Hospital 12 de Octubre (imas12)”, Universidad Complutense de Madrid, Madrid, Spain; 11 Department of Intensive Care, Department of Clinical Medicine, Copenhagen University Hospital – Rigshospitalet, University of Copenhagen, Copenhagen, Denmark; 12 Intensive Care Department, King Abdullah International Medical Research Center, King Abdulaziz Medical City, Ministry of National Guard Health - Affairs, and College of Medicine, King Saud Bin Abdulaziz University for Health Sciences, Riyadh, Saudi Arabia; 13 Department of Anaesthesiology, Critical Care and Pain, Tata Memorial Hospital, Homi Bhabha National Institute Mumbai, Mumbai, India; 14 Department of Anaesthesiology and Intensive Care Medicine, Division of Critical Care and Emergencies, Rikshopitalet Medical Centre, Oslo University Hospital, Oslo, Norway; 15 Department of Intensive Care Medicine, Ghent University Hospital, Gent, Belgium; 16 Department of Internal Medicine and Pediatrics, Faculty of Medicine and Health Sciences, Ghent University, Ghent, Belgium; 17 Department of Biomedical Sciences, Humanitas University, Via Levi Montalcini, Pieve Emanuele, MI Italy; 18 2IRCCS Humanitas Research Hospital, Via Manzoni 56, Rozzano, Milan 20089 Italy

## Abstract

**Purpose:**

To identify key components and variations in family-centered care practices.

**Methods:**

A cross-sectional study, conducted across ESICM members. Participating ICUs completed a questionnaire covering general ICU characteristics, visitation policies, team-family interactions, and end-of-life decision-making. The primary outcome, self-rated family-centeredness, was assessed using a visual analog scale. Additionally, respondents completed the Maslach Burnout Inventory and the Ethical Decision Making Climate Questionnaire to capture burnout dimensions and assess the ethical decision-making climate.

**Results:**

The response rate was 53% (respondents from 359/683 invited ICUs who actually open the email); participating healthcare professionals (HCPs) were from Europe (62%), Asia (9%), South America (6%), North America (5%), Middle East (4%), and Australia/New Zealand (4%). The importance of family-centeredness was ranked high, median 7 (IQR 6–8) of 10 on VAS. Significant differences were observed across quartiles of family centeredness, including in visitation policies availability of a waiting rooms, family rooms, family information leaflet, visiting hours, night visits, sleep in the ICU, and in team-family interactions, including daily information, routine day-3 conference, and willingness to empower nurses and relatives. Higher family centeredness correlated with family involvement in rounds, participation in patient care and end-of-life practices. Burnout symptoms (41% of respondents) were negatively associated with family-centeredness. Ethical climate and willingness to empower nurses were independent predictors of family centeredness.

**Conclusions:**

This study emphasizes the need to prioritize healthcare providers’ mental health for enhanced family-centered care. Further research is warranted to assess the impact of improving the ethical climate on family-centeredness.

## Introduction

Family-centered care in the ICU emphasizes family respect and dignity, promotes empathy and understanding, opens communication, collaboration, and shared decision-making between healthcare professionals (HCP) and the family members [1, 2]. This partnership improves health outcomes, patient and family experience of care, staff satisfaction, and resource utilization [3–5]. In a family-centered ICU, relatives are not visitors, they are potential partners, with their emotional and informational needs acknowledged and addressed [6]. Empowering family members and discussing with them patient’s preferences and values is the best way to recognize the role they play in the well-being and recovery of patients [7].

Barriers to patient and family-centered care in the ICU are organizational, individual, or interdisciplinary. Organizational barriers include high workload, visiting policies, and limited space and opportunities for privacy for families. Individual’s barriers include time constraints, competing priorities, difficult patients, familial distress, technological factors, HCP attitudes, personal challenges in engaging with families, and linguistic and cultural barriers. Moreover, interdisciplinary barriers involve conflicts and communication gaps among HCP at the workplace [8, 9]. Yet, the dedication to prioritizing family-centered care represents an additional obligation alongside the pressures of providing patient-centered care, resulting in increased workload and potential stress and burden [9]. Organizational resources for managing ethical conflicts, depersonalization (a component of burnout), and a sub-optimal ethical climate were reported as significant predictors of family-centered care [10].

We conducted a cross-sectional study involving members from the European Society of Intensive Care Medicine (ESICM). The primary aim was to describe and identify the variations in family-centered care practices across different regions worldwide. In each participating ICU, a single physician or a nurse documented local practices aimed at delineating the key components of family-centered care.

## Methods

ESICM affiliates (those registered on the ESICM mailing list, both members and non-members) received a message in July 2022 with a link inviting them to anonymously complete a questionnaire on family-centered care, work environment, ethical climate, and burnout. Two reminders were sent in August and September 2022, and participation in the study was allowed until December 2022. In each participating ICU, a single investigator (physician or a nurse) completed the questionnaire and checked that no one else from her/his ICU also completed the questionnaire. We also ensured that ICUs from a single region did not share identical characteristics, including country, number of hospital and ICU beds, and type of hospital. Online consent was obtained from all participants.

The data reported in tables referred to variables completed by the respondents and were collected online. The questionnaire included items identified from a literature review, previous experience, and semi-structured interviews with ICU-HCPs. The main components of the questionnaire included respondent’s characteristics, visitation policies, team-family interactions, and practices at the end of life. ICU-conflicts [11], symptoms of burnout [12], and the ethical decision-making climate questionnaire [13] were also collected. Conflicts were defined as a dispute, disagreement, incompatibility, opposition, or difference of opinion involving more than one individual and related to the patient’s management or to interpersonal conflict [11]. Symptoms of burnout were measured using the validated version of the 22-item Maslach Burnout Inventory (MBI, Human Services version) [12], which includes three subscales: emotional exhaustion (9 items), depersonalization (5 items), and personal accomplishment (8 items). Each item is scored from 0 (never) to 6 (every day). Respondents with high emotional exhaustion (≥ 27) and/or high depersonalization (≥ 10) scores were considered to have symptoms of burnout [14]. The ethical decision-making climate (EDMC) questionnaire included seven factors: not avoiding decision-making at end-of-life (EOL), mutual respect within the interdisciplinary team, open interdisciplinary reflection, ethical awareness, self-reflective physician leadership, active decision-making at end-of-life by physicians, and involvement of nurses in EOL [13, 15].

Visual analogue scales (VAS) were used to assess the intensity of unidimensional measures. Two anchors were provided: for 0 (no symptom/lowest rating) and 10 (the most intense symptom/highest rating). VASs are convenient, easy, and rapid to administer and have been provenproved reliable for measuring a characteristic, subjective phenomenon, or attitude that is believed to range across a continuum of values and cannot easily be directly measured [16, 17].

Family-centeredness was the primary endpoint of this study and was collected using a VAS with 0 indicating that the ICU was not at all family-centered and 10 indicating that family-centered care was a major priority for HCPs.

### Statistical analysis

The data were reported as medians and interquartile ranges (IQR), or numbers and percentages. Categorical variables were compared using Fisher’s exact test, and continuous variables were compared using the nonparametric Wilcoxon test or Kruskal-Wallis test. Spearman’s test was used to test correlations. Results are presented according to quartiles of family centeredness.

Factors independently associated with family centeredness were identified using a linear regression model. For all models, we first performed univariate analyses, including all the variables shown in Tables 1, 2 and 3. We then built a multivariate linear model with importance of family-centered care as the variable of interest. Variables yielding *p* < 0.20 in univariate analyses that were not considered a consequence of family-centered care were entered into the model. The final model was determined with a preplanned stepwise variable selection using an exit P value of 0.10. For linear models, the assumptions for linearity, independence, homoscedasticity, and lack of multicollinearity were carefully checked. The final model was a mixed linear model with region of the ICU entered as a random effect against the intercept. The model’s effectiveness was evaluated by assessing the percentage of variation (r2) it explained.

Splines and their 95%CI were constructed using the general additive model and then plotted.

All tests were two-sided, and P values less than 0.05 were considered statistically significant. Analyses were done using R software version 3.6.2 (https://www.r-project.org). Packages “lmer”, “lmerTest”, and “mgcv” were used for this analysis.

## Results

An invitation to complete the questionnaire was sent to 66,654 ESICM-affiliates working in 8041 hospitals. The email was received by individuals working in 1486 ICUs, including 683 who opened it. Overall, 359 questionnaires were completed, leading to a response rate of 53% (24% of all ICUs).

Among the participating ICUs, 223 (62%) were located in Europe (19 in Eastern Europe, 115 in Northern Europe, and 89 in Southern Europe), 33 (9%) in Asia, 21 (6%) in South America, 18 (5%) in North America, 15 (4%) in the Middle East, and 13 (4%) in Australia/New Zealand. The location was missing for 36 (10%) participants.

As shown in Tables 1 and 297 (83%) respondents were physicians and 62 (17%) were nurses, working in university-affiliated hospitals in 62% of cases, and having an ICU experience of 13 (8–21) years. Family members were allowed to visit the patient 6 (2–22) hours per day, 54% were allowed to visit the patient at night, and 29% allowed family members sleep in the ICU. A family information leaflet was delivered by 212 (59%) ICUs, including 48 ICUs having a digital leaflet. As shown in Table 2, a waiting room was available in 86% of the participating ICUs, but was described as only moderately welcoming (5/10 [3–7]), comfortable (5/10 [2–7]), or adapted (4/10 [2–7]). A receptionist was present in 122 (34%) ICUs. A room dedicated to family members was available in 148 (41%) ICUs, and a dedicated bathroom was available in 165 (46%) ICUs. Wi-Fi was available in 195 (54%) ICUs, and a coffee machine in 135 (38%) ICUs. Less than half the ICUs had a nurse facilitator (*n* = 64, 18%) or a dedicated psychologist (*n* = 131, 36%). A social worker was present in 208 (58%) ICUs.

**Table 1:** Respondent’s characteristics according to the quartile of ranking of family centeredness

Responses (*N* = 359) [Median (IQR) or Numbers (%)]	First quartile, *N* = 127 (35.4%)	Second quartile, *N* = 65 (18.1%)	Third quartile, *N* = 84 (23.4%)	Fourth quartile, *N* = 83 (23.1%)	*P* Value
**Role in the ICU**					0.22
*Physicians*	99 (88)	52 (80)	73 (86,9)	73 (88)	
*Nurses*	28 (22.0)	13 (20.0)	11 (13.1)	10 (12.0)	
**Age**	45 (37–53)	47 (43–57)	47 (41–53)	51 (43–59)	0.04
**Female sex**	56 (44.1)	32 (49.2)	34 (40.4)	32 (38.6)	0.81
**ICU experience (month)**	150 (60–247)	192 (120–246)	130 (78–240)	196 (120–312)	0.01
**Hospital characteristics**					
Work in a university affiliated hospital	63 (62.4)	35 (63.6)	43 (61.4)	39 (60.0)	0.93
Number of hospital beds	450 (200–800)	450 (215–999)	400 (252–700)	400 (200–760)	0.70
Number of ICU beds	15 (10–25)	20 (12–30)	18 (12–30)	15 (11–20)	0.13
Number of nurses	2 (2–3)	2 (2–2)	2 (1–2)	2 (1–2)	0.04
Number of physicians	8 (4–12)	9 (5–12)	12 (8–16)	9 (6–12)	0.01
Number of residents	4 (2–8)	5 (2–9)	6 (3–12)	6 (3–10)	0.01
Number of ICU admissions in 2019	700 (350–1200)	800 (438–1327)	600 (400–1100)	742 (436–1000)	0.85
ICU Morality rate in 2019	16 (10–25)	15 (10–20)	17 (11–27)	18 (8–25)	0.61
**Perceived conflicts over the last month** ɸ					
Within the nursing staff	3 (2–5)	3 (2–4)	3 (2–4)	2 (1–3)	0.05
Within the medical staff	3 (2–6)	3 (2–5)	3 (2–5)	3 (2–4)	0.35
With family members	2 (1–4)	3 (2–4)	2 (1–3)	2 (1–3)	0.34
**Maslach Burnout inventory**					
Score for emotional exhaustion	21 (12–28)	16 (9–27)	17 (10–26)	15 (7–21)	0.008
Score for depersonalization	8 (4–12)	7 (3–11)	7 (4–11)	3 (2–8)	0.0007
Score for personal accomplishment	31 (26–36)	33 (28–38)	33 (29–39)	37 (32–40)	< 0.0001
**Ethical Decision-Making Climate score**	99 (90–118)	117 (109–124)	122 (110–129)	128 (119–137)	< 0.0001

ɸ Visual analogue scales were used to assess the intensity of unidimensional measures. Two anchors were provided to family members for 0 (no symptom/lowest rating) and 10 (the most intense symptom/highest rating)

**Table 2:** Visitation policies and team-family interactions according to the quartile of ranking of family centeredness

Responses (*N* = 359) [Median (IQR) or Numbers (%)]	First quartile, *N* = 127 (35.4%)	Second quartile, *N* = 65 (18.1%)	Third quartile, *N* = 84 (23.4%)	Fourth quartile, *N* = 83 (23.1%)	*P* Value
**Visitation policies**					
Visiting hours (median [IQR])	3.00 [2.00, 10.00]	8.00 [2.00, 22.00]	11.00 [4.00, 24.00]	8.00 [3.00, 24.00]	< 0.001
A waiting room is available for family members (%)	94 (74.0)	58 (89.2)	80 (95.2)	78 (94.0)	< 0.001
Number of synchronous visitors allowed	2.00 [1.00, 2.00]	2.00 [2.00, 2.00]	2.00 [2.00, 2.25]	2.00 [2.00, 2.00]	0.010
Family visits allowed at night (%)	46 (36.2)	41 (63.1)	50 (59.5)	57 (68.7)	< 0.001
Family members are allowed to sleep in the ICU (%)	13 (10.2)	21 (32.3)	30 (35.7)	41 (49.4)	< 0.001
Children visits are allowed (%)	69 (54.3)	43 (66.2)	64 (76.2)	60 (72.3)	0.005
A specific room is available for family members	35 (27.6)	32 (49.2)	49 (58.3)	32 (38.6)	< 0.001
**A Family information leaflet is delivered at admission (%)**	65 (52.2)	41 (63.1)	57 (67.9)	69 (83.1)	< 0.001
**Routine use of ICU diaries**	27 (21.3)	19 (29.2)	20 (23.8)	23 (27.7)	0.277
**Team-family interactions**					
A nurse facilitator is available for family members (%)	16 (12.6)	6 ( 9.2)	16 (19.0)	26 (31.3)	0.001
A psychologist / a social worker are available (%)	42 (33.1)/65 (51.2)	25 (38.5)/ 43 (66.2)	29 (34.5)/ 50 (59.5)	46 (65.4)/ 50 (82.3)	0.01/0.05
Information is given (%)					< 0.001
*Once a day / Several times a day*	97 (76) / 16 (13)	37 (57) / 16 (25)	43 (51) / 31 (37)	36 (43) / 40 (48)	
*2–3 times a week*	11 (9)	9 (14)	8 (9.5)	6 (7)	
*No response*	3 ( 2.4)	3 ( 4.6)	2 ( 2.4)	1 ( 1.2)	
Routine family conference after 3 days of ICU admission (%)	37 (29.1)	32 (49.2)	44 (52.4)	57 (68.7)	< 0.001
Who provides information to family members? (%)					0.020
*The senior physician in charge*	51 (40.2)	21 (32.3)	30 (35.7)	41 (49.4)	
*The senior physician in charge and the physician in training*	38 (29.9)	33 (50.8)	40 (47.6)	31 (37.3)	
*The physician in training*	19 (15.0)	9 (13.8)	6 ( 7.1)	5 ( 6.0)	
*The same senior physician for all families*	9 ( 7.1)	1 ( 1.5)	6 ( 7.1)	3 ( 3.6)	
*The physician on call*	10 ( 7.9)	1 ( 1.5)	2 ( 2.4)	3 ( 3.6)	
Nurses are always present during family interviews (%)	56 (44.1)	38 (58.5)	52 (61.9)	56 (67.5)	< 0.001
Videoconferences are part of routine interaction (%)	61 (48.0)	38 (58.5)	57 (67.9)	70 (84.3)	< 0.001
Nurse empowerment is a priority in the ICU (%)	52 (40.9)	24 (36.9)	45 (53.6)	57 (68.7)	< 0.001
Family members are not allowed to attend rounds (%)	105 (82.7)	42 (64.6)	43 (51.2)	38 (45.8)	< 0.001
Family members are not allowed to participate to care (%)	49 (38.6)	15 (23.1)	19 (22.6)	13 (15.7)	< 0.001
Family members are not allowed to be present during CPR (%)	99 (78.0)	40 (61.5)	45 (53.6)	45 (54.2)	< 0.001
Family empowerment is a priority in the ICU (%)	88 (69.3)	58 (89.2)	76 (90.5)	75 (90.4)	< 0.001

Respondents ranked family-centered as very important (rate of 7 (6–8)/10).

Family meetings with the ICU team occurred at least once a day in 88% of the ICUs, and in a dedicated room in 232 (65%) ICUs. Information was also delivered remotely by smartphone-based videoconference or short message service in 99 (28%) ICUs. Information was delivered by the senior physician in 80% of the cases, either alone (143 ICUs, 40%), or accompanied by the physician in training (142 ICUs, 40%). Respondents reported that nurses were always present during family interviews in 56% of the ICUs. A day-3 family meeting was delivered routinely in 170 (47%) ICUs, to which nurse presence was routine in 56% of the cases. 297 (83%) respondents reported empowering family members as a priority, whereas empowering nurses was a priority for 178 (50%) respondents.

Relatives were not allowed to attend ICU rounds in 228 (63.5%) ICUs. They also were not allowed to witness cardiopulmonary resuscitation (229 ICUs, 63.5%).Moreover, in only 96 (27%) ICUs, relatives were not allowed to participate in patient care.

Table 3 shows end-of-life practices in the participating ICUs as reported by the respondent. Family members could initiate end-of-life discussions in 67 (19%) ICUs. The level of involvement of family members in end-of-life decisions was 8 (6–9)/10. The most commonly observed decision-making model was the shared decision-making approach (234 ICUs, 65%), with the decision being made mostly by the family members in 76 (21%) ICUs and mostly by physicians in 17 (5%) ICUs.

**Table 3:** End of life decision making and interaction with family members

Responses (*N* = 359) [Median (IQR) or Numbers (%)]	First quartile, *N* = 127 (35.4%)	Second quartile, *N* = 65 (18.1%)	Third quartile, *N* = 84 (23.4%)	Fourth quartile, *N* = 83 (23.1%)	*P* Value
**Who can initiate end of life decisions?**					0.234
*Only physicians*	86 (67.7)	44 (67.7)	52 (61.9)	52 (62.7)	
*Physicians and nurses*	20 (15.7)	15 (23.1)	20 (23.8)	18 (21.7)	
*Family members*	17 (13.4)	13 (20.0)	20 (23.8)	17 (20.5)	
**Decisions to withhold/withdraw life sustaining therapies involve**					0.004
*Only ICU physicians*	51 (40.2)	18 (27.7)	22 (26.2)	20 (24.1)	
*ICU physicians and ICU nurses*	60 (47.3)	44 (67.7)	58 (69)	58 (69.8)	
**Psychologists are involved in the decisions**	8 ( 6.3)	7 (10.8)	7 ( 8.3)	19 (22.9)	0.002
**Palliative care consultants are involved in the decisions**	5 ( 3.9)	15 (23.1)	11 (13.1)	20 (24.1)	< 0.001
**Family involvement in end of life decisions** ɸ	6.00 [4.00, 7.00]	8.00 [6.00, 9.00]	8.00 [7.00, 9.00]	9.00 [7.00, 10.00]	< 0.001
**Family involvement in end of life decisions**					0.007
*Family members are not involved and not informed*	2 ( 1.6)	1 ( 1.5)	3 ( 3.6)	0 ( 0.0)	
*Family members are not involved but are informed*	10 (7.9)	2 ( 3.1)	3 ( 3.6)	2 ( 2.4)	
*Family members and healthcare providers share the decision*	80 (63)	42 (64.6)	59 (70.2)	53 (63.8)	
*Family members make the decision*	21 (16.5)	17 (26.2)	15 (17.9)	23 (27.7)	
*No answer*	14 (11.0)	3 ( 4.6)	4 ( 4.8)	5 ( 6.0)	
**Routine end of life family conferences**	30 (23.6)	42 (64.6)	50 (59.5)	53 (63.9)	< 0.001
**Routine delivery of bereavement information leaflet**	32 (25.2)	31 (47.7)	37 (44.0)	27 (32.5)	0.07
**Palliative care consultants attend the conference**	10 ( 7.9)	16 (24.6)	13 (15.5)	19 (22.9)	0.005
**Psychologists / social workers attend the conference**	15 (11.8) / 9 ( 7.1)	9 (13.8) / 11 (16.9)	8 ( 9.5) / 9 (10.7)	23 (27.7) / 13 (15.7)	0.004/0.12
**Routine delivery of spiritual care**	76 (59.8)	48 (73.8)	63 (75.0)	65 (78.3)	0.11
**Routine delivery of palliative care by external consultants**	10 (7.9)	14 (21.5)	15 (17.9)	18 (21.7)	0.003
**Routine request of an ethical consultant**	4 ( 3.1)	2 ( 3.1)	7 ( 8.3)	7 ( 8.4)	0.05
**Routine family meeting immediately after patient’s death**	40 (31.5)	34 (52.3)	43 (51.2)	46 (55.4)	0.04
**A condolence letter is routinely sent to family members**	11 ( 8.7)	11 (16.9)	16 (19.0)	9 (10.8)	0.21
**Post death debriefing is offered to family members**	19 (15.0)	16 (24.6)	24 (28.6)	25 (30.1)	0.07
**Less than 10% of doctors are trained in communication**	52 (40.9)	18 (27.7)	24 (28.6)	14 (16.9)	0.04
**Less than 10% of nurses are trained in communication**	56 (44.1)	20 (30.8)	20 (23.8)	20 (24.1)	< 0.0001

ɸ Visual analogue scales were used to assess the intensity of unidimensional measures. Two anchors were provided to family members for 0 (no symptom/lowest rating) and 10 (the most intense symptom/highest rating)

End-of-life family conferences were routine in 175 (49%) ICUs. More than one-third (127, 36%) ICUs routinely delivered bereavement information leaflets, and family meetings immediately after patient’s death were made in 163 (45%) ICUs, whereas post-death debriefing was offered in only 84 (23%) ICUs.

Symptoms of burnout were reported in 115 of the 279 respondents (41%, 80 respondents did not fully complete the MBI). The global score of the Ethical Decision-Making Climate was 118 (102–129).

As shown in tables and figures, Table 2 reports significant differences in visitation policies and team-family interactions according to quartiles of family-centeredness. Visiting hours and the possibility of visiting the patient at night or sleeping in the ICU increased exponentially (doubled or almost tripled) with the importance given to family-centered care. Availability of a waiting room or a room specifically dedicated to family members was also twice as frequent where scores for family-centeredness were higher, as was the provision of a family information leaflet. At least daily information, routine day-3 conferences, and the willingness to empower nurses and include them in family interviews also significantly increased with higher family-centeredness. Respondents ranking the importance of family-centered care higher, significantly more frequently allowed family members to attend the rounds, participate in patient care, or witness CPR. With regard to end-of-life decision-making, the involvement of the nursing staff and other allied professionals significantly increased alongside the importance of family-centered care, end-of-life family conferences, meeting with the family immediately after the death in the ICU and post-death debriefing were significantly more frequent with increasing family-centeredness.

**Fig. 1:**
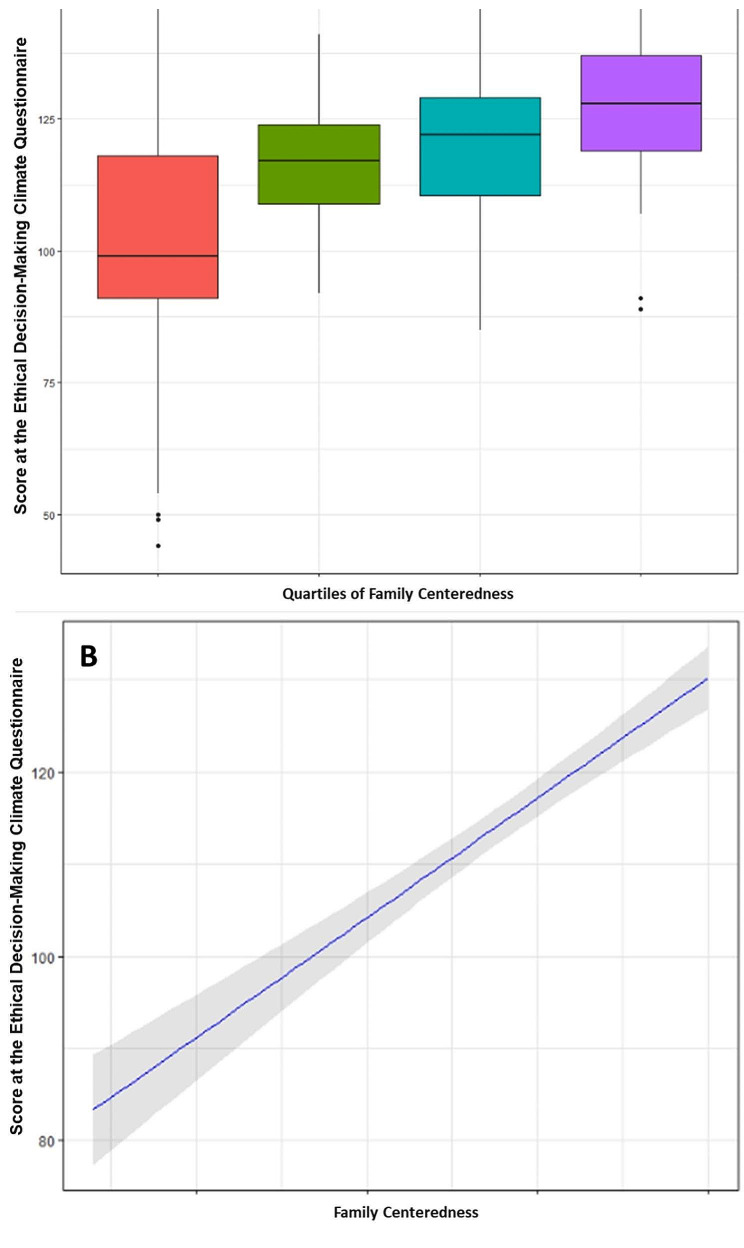
Ethical Decision Making Climate Questionnaire Score as a function of quartiles (Panel A) or continuous values (Panel B) of family centeredness. A visual analogue scale was used to assess the intensity of family centeredness. Two anchors were provided to family members for 0 (family centered care is not all a priority in our ICU) and 10 (family centered care is a major priority in our ICU)

**Fig. 2:**
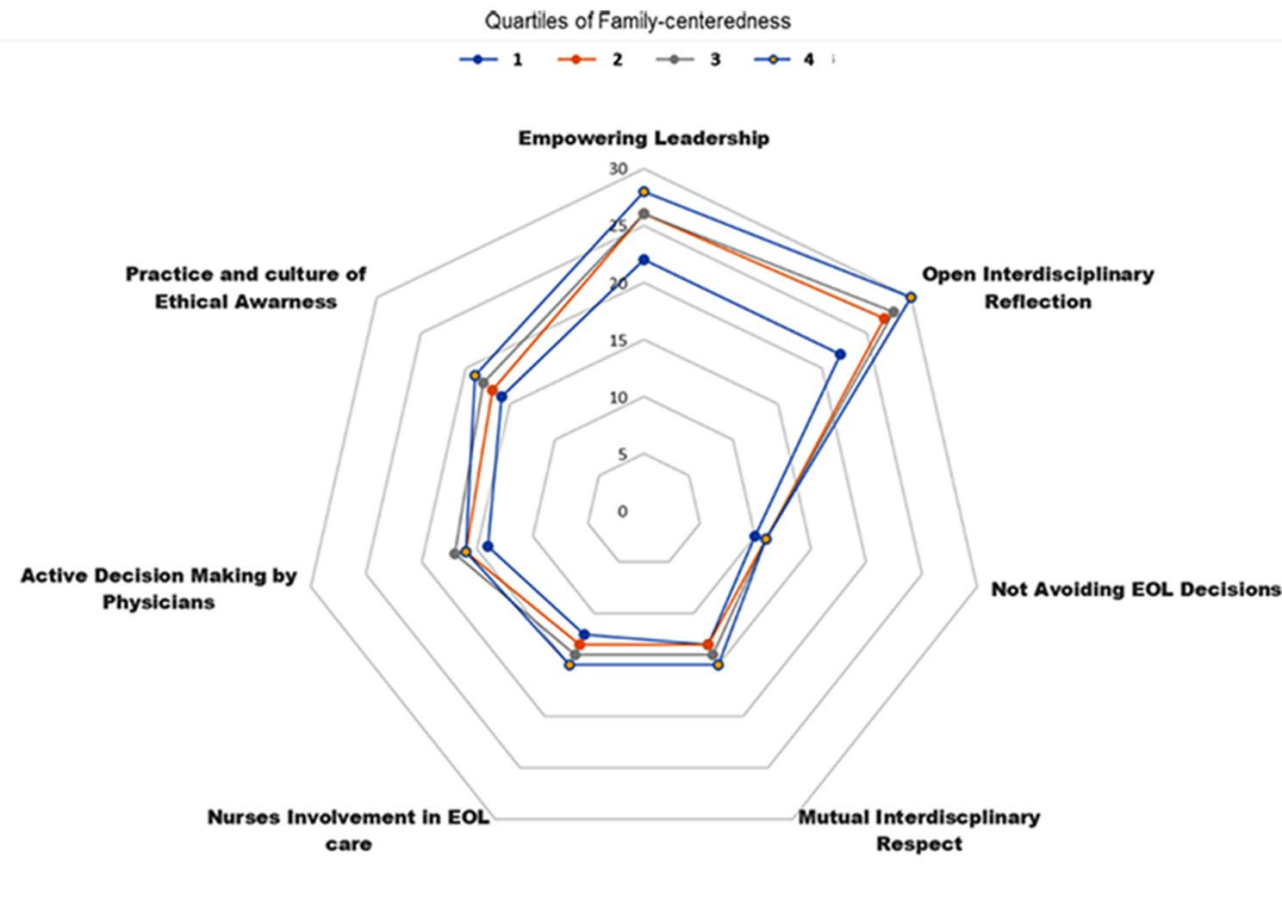
Median score of each of the seven distinct ethical climate factors part of the Ethical Decision Making Climate Questionnaire according to quartiles of family centeredness. Larger values of each factor indicate a more positive environment for decision making. A visual analogue scale was used to assess the intensity of family centeredness. Two anchors were provided to family members for 0 (family centered care is not all a priority in our ICU) and 10 (family centered care is a major priority in our ICU)

**Fig. 3:**
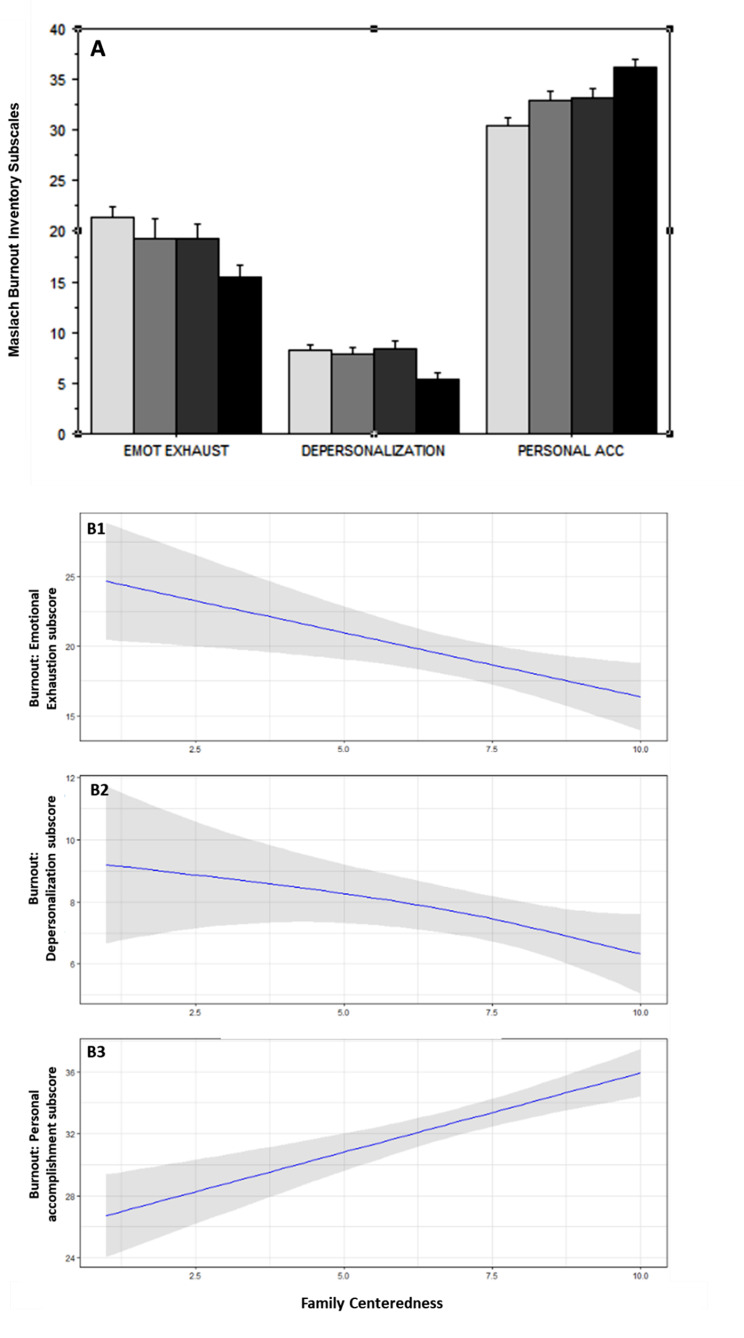
Maslach Burnout Inventory (MBI) Scale and family centeredness. The three MBI domains, namely, emotional exhaustion, depersonalization, and personal accomplishment, are displayed according to quartiles of family centeredness in Panel A (clear gray bars for the lower quartile, black bars for the higher quartile). In Panel B1, B2 and B3, the three MBI domains are displayed according to continuous values of family centeredness. A visual analogue scale was used to assess the intensity of family centeredness. Two anchors were provided to family members for 0 (family centered care is not all a priority in our ICU) and 10 (family centered care is a major priority in our ICU)

As shown in Fig. 1, the EDMC score increased significantly with increased family-centeredness. Figure 2 illustrates each of the seven domains of the EDMC were significantly associated with increased family-centeredness. Figure 3 shows the score in each of the three domains of the MBI was significantly associated with family-centeredness. Symptoms of burnout were present in 53% of the respondents in the lower family-centeredness quartile, as compared to 24% in the higher family-centeredness quartile (*P* < 0.0001).

By multivariable analysis, the EDMC score (Estimate per point 0.06; 95%CI 0.05–0.07, *P* < 0.001) and the willingness to empower nurses (Estimate 0.48; 95%CI 0.05–0.91, *P* = 0.03) were independent predictors of the importance of family centeredness. Random effect for the different regions was not associated with the primary outcome variable in this model.

## Discussion

This cross-sectional study describes the key components of family-centered care, which not only enhances quality of care but also provides a supportive environment that offers a more empathetic and humanized ICU experience to family members [2, 18]. This study emphasizes the pivotal role of family-centered care acknowledging the emotional and informational needs of family members, and in shaping various dimensions of ICU practice. Notably, the positive correlation between family-centeredness and key elements such as visitation policies, team-family interactions, end-of-life discussions, a higher frequency of family meetings and remotely-delivered information suggest that fostering an environment where families are actively involved positively impacts communication strategies [19].

Identifying and addressing barriers to family-centered care is crucial for successful implementation. The study emphasizes the impact of organizational resources on family-centered care, especially in managing conflict and preventing burnout among healthcare professionals. This finding highlights the need for healthcare organizations to adopt systemic approaches, integrating family-centered care into their core values providing necessary resources to mitigate barriers. Strategies could include training programs, support systems, and ethical decision-making frameworks to create an environment conducive to family-centered care [20, 21].

The inclusion of ICUs from diverse global regions provides a nuanced understanding of family centered-care’s universality. Despite cultural and system differences, the study suggests the importance of family-centered care is recognized globally [22, 23].

The study’s identification of burnout symptoms among healthcare professionals and its’ correlation with family-centeredness illustrates the interdependence between HCP mental health and patient and family-centered care [24]. Acknowledging the potential impact of family-centered care on the well-being of healthcare professionals, if confirmed, emphasizes the importance of a holistic approach to care delivery [25]. Furthermore, the positive correlation between family-centered care and the Ethical Decision-Making Climate suggests a potential pathway for enhancing ethical practice in the ICU through prioritizing family-centeredness [15].

The study has certain limitations, such as its cross-sectional design and reliance on self-reported data. Future research could employ longitudinal approaches and objective measures to establish causation and strengthen the evidence base. A substantial limitation of this study is its response rate and that each participating ICU had only one representative completing the questionnaire, creating a potential bias when reporting personal opinion or experience (i.e., nurses vs. physicians or individual burnout). Furthermore, allied HCPs are very little represented, and senior physicians with extensive ICU experience highly represented. This may not fully capture the collective experiences and perspectives of the entire healthcare team [26]. Additionally, despite including ICUs from various global regions, the study lacks a fully representative sample of ICUs in each region. Consequently, it is not possible to definitively assert the absence of significant variations in family-centered care across the participating countries. Nevertheless, the inclusion of various levels of ICUs, is likely to cover the diverse spectrum of practices across the globe. Additionally, strategies to assess the long-term effects of enhanced family-centered care on patient and family outcomes could be explored. Another inherent limitation of these surveys is that the reliability of the individual responses cannot be ensured.

In conclusion, this cross-sectional study = suggests the need to integrate family-centered care into ICU practices globally. By addressing organizational barriers, acknowledging the impact on healthcare professionals’ well-being, and recognizing the correlation with ethical decision-making, healthcare systems can strive for a more comprehensive and compassionate approach to critical care. As the healthcare landscape evolves towards patient-centered models, embracing family-centered care emerges as a key component for achieving holistic and empathetic care in critical settings.

## Data Availability

All data are available on request to ESICM.
